# EEG Microstate Analysis in Drug-Naive Patients with Panic Disorder

**DOI:** 10.1371/journal.pone.0022912

**Published:** 2011-07-29

**Authors:** Mitsuru Kikuchi, Thomas Koenig, Toshio Munesue, Akira Hanaoka, Werner Strik, Thomas Dierks, Yoshifumi Koshino, Yoshio Minabe

**Affiliations:** 1 Department of Psychiatry and Neurobiology, Graduate School of Medical Science, Kanazawa University, Kanazawa, Japan; 2 Department of Psychiatric Neurophysiology, University Hospital of Psychiatry, University of Bern, Bern, Switzerland; 3 Research Center for Child Mental Development, Kanazawa University, Kanazawa, Japan; Rikagaku Kenkyūsho Brain Science Institute, Japan

## Abstract

Patients with panic disorder (PD) have a bias to respond to normal stimuli in a fearful way. This may be due to the preactivation of fear-associated networks prior to stimulus perception. Based on EEG, we investigated the difference between patients with PD and normal controls in resting state activity using features of transiently stable brain states (microstates). EEGs from 18 drug-naive patients and 18 healthy controls were analyzed. Microstate analysis showed that one class of microstates (with a right-anterior to left-posterior orientation of the mapped field) displayed longer durations and covered more of the total time in the patients than controls. Another microstate class (with a symmetric, anterior-posterior orientation) was observed less frequently in the patients compared to controls. The observation that selected microstate classes differ between patients with PD and controls suggests that specific brain functions are altered already during resting condition. The altered resting state may be the starting point of the observed dysfunctional processing of phobic stimuli.

## Introduction

Panic disorder (PD) is a common mental disorder [1]–[3], which is thought to involve aberrant cognitive features such as catastrophic misinterpretation of bodily sensations even during the inter-attack conditions [4]. The lifetime prevalence of PD with or without agoraphobia is about 3–4% [5]. PD is associated with other psychiatric disorders [1]–[2] and a high risk of suicidal attempts [6]. Recent studies emphasize the importance of panic syndromes as a significant source of disability in the general population [2], [7]. It has been argued that the pathophysiology of PD results from dysfunctions in neural microcircuits such as fronto-temporo-limbic circuits [8]-[9]. A number of neuroimaging studies have demonstrated aberrant brain activity in these cortical areas not only during panic attacks [10]–[11] but also during resting [12]–[17]. Furthermore, factor analyses of functional connectivity of spontaneous fMRI-BOLD signal fluctuations, which yields so-called resting-state networks [18]–[24] demonstrated aberrant RSNs in patient with anxiety disorders [25]–[28].

The observation that resting state brain activity can efficiently be parsed into a small and consistent set of patterns that have network characteristics has been made in EEG data, too. These patterns transiently synchronized EEG measurements have been referred to as microstates, and their rationale is briefly outlined here: Traditionally, spontaneous EEG analysis relies mainly on the power variation in different frequency bands; however, observing this variation inherently sacrifices temporal accuracy due to the time-frequency uncertainty principle. To account for short-lasting fluctuations of neuronal activity, analysis methods in the time domain are required. The momentary scalp EEG field is a direct measure of the momentary global state of the brain. It represents the summation of all concurrently active sources in the brain irrespective of their frequency [29]–[32]. A series of momentary scalp EEG field remains quasi-stable for periods of about 50–120 ms; such stability is not found in amplitude and power modulations at the single electrodes. During these periods of quasi-stability, the topography remains fixed, while polarity can invert; such inversions are driven by oscillations of the dominant generators. These periods of stable EEG topography are referred to as EEG microstates [30]. Interestingly, the scalp topographies observed at rest can be clustered into a limited number of map classes with prototypical configurations, and four topographies typically are sufficient to explain about 80% of the variance [29], [32]. Recent studies using simultaneous EEG and fMRI recording demonstrated that EEG-defined microstates correlate significantly with RSNs assessed by fMRI [18], [33] and the four most typical microstates of spontaneous EEG seem to be associated with four of the fMRI based RSNs [18].

A number of studies applied spontaneous EEG microstate analysis to assess changes in global brain coordination in association with spontaneous thoughts [34] or brain maturation [29]. In addition, alterations of microstates have been reported in schizophrenia [35]–[37], depression [38], and Alzheimer’s disease [39]-[40]. However, no previous study analyzed resting state EEG in anxiety disorder as a function of spatially separate microstate classes.

In this study, we hypothesized that aberrant brain resting state networks in patients with PD must be reflected by deviant EEG microstate properties. In order to test this hypothesis, we evaluated EEG microstates in drug- naïve patients with PD in comparison with normal controls.

## Materials and Methods

### Subject

The patient group consisted of 11 men and 7 women who consulted the psychiatric outpatient clinic of Kanazawa University Hospital. All were diagnosed by trained psychiatrists, and fulfilled DSM-IV criteria for PD with or without agoraphobia. Mean age (±SD) was 30.2±9.1 years (range, 16–52), the mean duration of disease (±SD) was 57.7±97.0 weeks (range, 1.5–380). None of the patients had any comorbidity with other psychiatric disorders, such as major depression. Patients had never received psychoactive medication (e.g., antipsychotics, anticholinergics, antidepressants, anticonvulsants, anxiolytics, cerebral metabolic activators, or cerebral vasodilators). The current severity (mild, moderate, severe, and in partial remission) of panic attacks was specified for each patient according to the DSM-III-R criteria.

Control subjects consisted of 18 healthy volunteers (11 men and 7 women), aged (±SD) 30.6±9.8 years (range, 20–54). The control group was not significantly different from the PD group in age or gender. They were staff members of Kanazawa University Hospital or their family members, and did not have any personal or family history of psychiatric or neurological disease, which was confirmed both by a self-reported past history and by a psychiatric examination of present mental state using the DSM-IV criteria of axis I. All were functioning normally and independently in their daily lives. All subjects in the two groups were right-handed. The present study was approved by the Ethics Committee of Kanazawa University, and written informed consent was obtained from all participants.

### EEG recording

Subjects were recorded while lying in a soundproof, light-controlled recording room. Electrodes were attached to the scalp with paste at the following positions of the International 10–20 System: Fp1, Fp2, F3, F4, Fz, F7, F8, C3, C4, P3, P4, Pz, T5, T6, O1, and O2, reference was linked earlobes. Eye-movements were monitored with an additional EOG channel. Electrode-impedances were kept below 5 kΩ. The EEG was filtered (1.5 to 60 Hz bandpass), amplified, digitized (200 Hz) and digitally stored using an 18-channel electroencephalograph (EEG-44189, Nihon Kohden, Tokyo, Japan). All subjects were instructed to relax and keep their eyes closed throughout the recording period. The subjects were observed via a video monitoring system. When they appeared to become drowsy, they were asked to briefly open their eyes and remain awake, after which the EEG recording was continued. From the recorded data, epochs of 2.56 sec (512 data points) duration recorded in the eyes-closed but awake state were selected based on visual inspection of EEG and EOG recordings. Epochs containing eye movements, blinks, muscle activities or other artifacts were excluded. The number of available epochs per subject ranged from 18 to 61. All epochs were recomputed against common average reference.

### EEG microstate analysis

The microstate analysis followed the standard procedure used in earlier work [29]. The selected EEG epochs were digitally band pass filtered from 2–20 Hz. Global Field Power (GFP), which quantifies the overall potential variance across the set of electrodes, was computed at each sample in time. Since topography remains stable around peaks of GFP and changes during the troughs, only topographies at momentary maxima of the GFP were further analyzed. As in previous work [36], four optimally fitted microstate class topographies were computed using a modified version of the K-mean clustering algorithm [41]. The algorithm searched four classes of microstate topography and assigned each EEG topography to one of these classes. This number of classes has previously been found to be optimal and was maintained for compatibility with the existing literature. Microstate class topographies were computed individually and averaged across subjects, using a permutation algorithm that maximized the common variance over subjects [36].

Within each subject, microstates were identified as continuous epochs within which all topographies were assigned to the same class. No minimal microstate duration was required. Individual microstate profiles were computed for each class, consisting of mean microstate duration (“duration”), mean number of microstates per second (“occurrence”) and percentage of total analysis time occupied in that state (“percent total time”). For each microstate measure, a two way ANOVA was performed with subject group as between, and microstate class as within subject factors. Significant results including group were post hoc tested using unpaired, double-ended t-tests. Relationships between each microstate measure and the current severity (in partial remission  = 1, mild = 2, moderate  =  3 and severe  =  4) of panic attacks according to the DSM-III-R criteria was assessed using Spearman's rank correlation coefficient. Statistical significance was defined as p<0.05.

The microstate syntax was assessed as follows: For each subject, the number of transitions from each of the four classes to any other class was counted; these numbers were normalized to fractions of all between-class transitions of the subjects. Given four classes, were thus obtained for each subject twelve values for all possible sequences doublets, and 36 values for sequence triplets. For each transition value, a double-ended t-test was performed between groups.

## Results

### EEG microstate analysis

**Figure 1:**
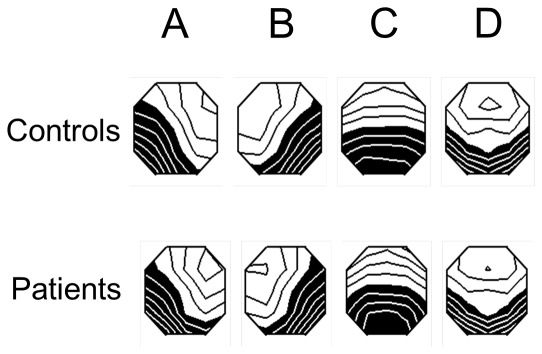
Microstate classes of patients and controls. Mean normalized equipotential maps of the four microstate classes (A–D) of patients and controls. Using a linear scale, the map areas of opposite polarity are arbitrarily coded in black and white.

**Figure 2:**
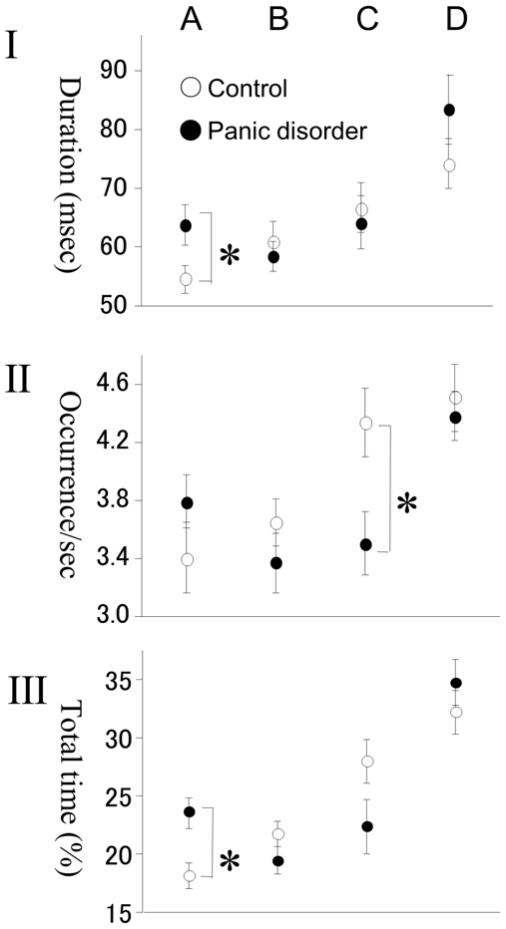
Microstate statistics. Duration (I), occurrence/second (II), and percent total time covered (III), of the 4 microstate classes (A–D) of controls (n = 18; open circle) and drug naïve patients with panic disorder (n = 18; closed circle). Values indicate means ± S.E. * p<.05: comparison between patients (n = 18) and controls (n = 18) using double-ended t-test.

The group mean maps of the 4 microstate classes are shown in Fig. 1. They closely resembled classes previously observed in eye-closed EEG [29] and were labeled accordingly. Both groups had two microstate classes (A and B) with diagonally oriented axes of the mapped field, one class (C) with a clear anterior-posterior orientation, and one (D) with a fronto-central extreme location. The four microstate classes accounted for a mean of 76.2% (S.D.: 6.9%) of the data variance across healthy controls, and of 75.7% (S.D.: 7.2%) across patients with PD. The duration, occurrence, and total time for the 4 microstate classes are shown in Fig. 2.

Microstate mean duration varied between 54.5 and 83.4 ms in patients and controls for the different microstate classes. The group × microstate class ANOVA showed no main group effect, but a significant interaction between group and microstate class (F = 2.75; df = 3,108; p = .046). This was due to significantly longer microstates of class A of patients compared to controls (t = 2.20, df = 34, p = .035) (Table 1, Fig 2A).

**Table 1:** Mean duration of microstate (SD) in patients with panic disorder (n = 18) and normal controls (n = 18).

	Panic disorder (ms)	Control (ms)	t value	P value (2-tailed t-test)
Class A	63.81 (14.71)	54.55 (10.14)	2.20	.035
Class B	58.47 (10.47)	61.03 (13.75)	−.63	n.s.
Class C	64.21 (18.92)	66.63 (18.17)	−.39	n.s.
Class D	83.40 (25.34)	74.14 (17.62)	1.27	n.s.

Microstate occurrence ranged between 3.37 and 4.51 microstates/second. The ANOVA showed no main group effect, but a significant interaction between group and microstate class (F = 7.10; df = 3,108; p<.0.01). This was because microstates of class C were significantly less frequent in patients compared to controls (t = -2.58, df = 34, p = .015) (Table 2, Fig 2B).

**Table 2:** Mean occurrence of microstate (SD) in patients with panic disorder (n = 18) and normal controls (n = 18).

	Panic disorder (/sec)	Control (/sec)	t value	P value (2-tailed t-test)
Class A	3.79 (.78)	3.41 (1.05)	1.27	n.s.
Class B	3.37 (.85)	3.65 (.68)	−1.10	n.s.
Class C	3.51 (.86)	4.34 (1.02)	−2.58	.015
Class D	4.38 (.73)	4.51 (.96)	−.45	n.s.

Percent of total time covered by the different microstate classes ranged between 18.2% and 34.7%. The ANOVA showed a significant interaction between group and microstate class (F = 4.00 df = 3,108; p = .010) that was due to significantly more percent total time in microstates of class A in patients than controls (t = 3.21, df = 34, p = .003) (Table 3, Fig 2C).

**Table 3:** Mean Total time (%) of microstate (SD) in patients with panic disorder (n = 18) and normal controls (n = 18).

	Panic disorder (%)	Control (%)	t value	P value (2-tailed t-test)
Class A	23.5 (5.4)	18.2 (4.6)	3.21	.003
Class B	19.9 (5.1)	21.7 (4.7)	−1.38	n.s.
Class C	23.3 (9.7)	28.0 (7.9)	−1.91	n.s.
Class D	34.7 (8.2)	32.2 (7.9)	.95	n.s.

Between any microstate measures above and the current severity of panic attacks according to the DSM-III-R criteria, there was no significant correlation (*P*>0.05).

### EEG microstate syntax

**Figure 3:**
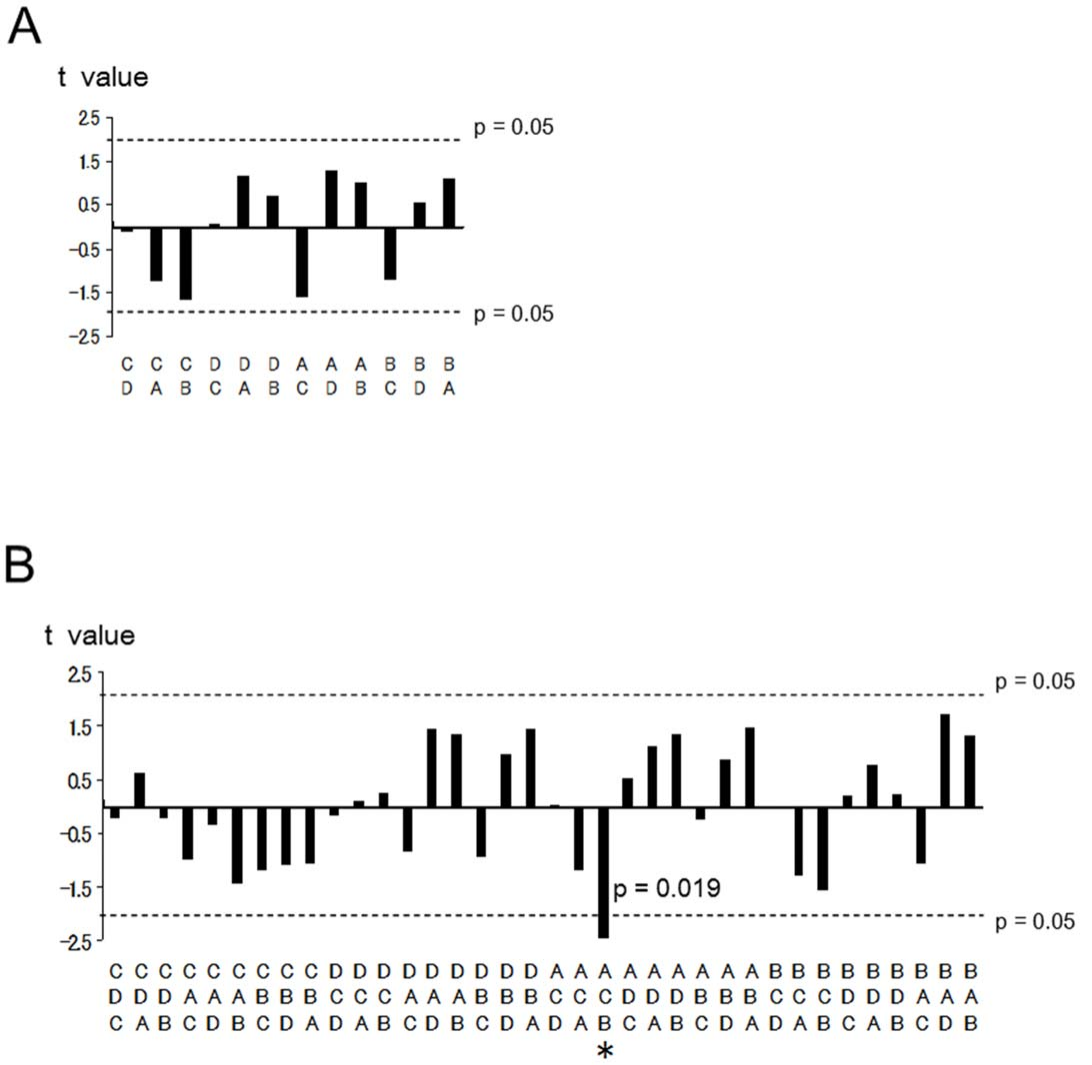
Microstate syntax. (A) T values (unpaired t-test) between healthy controls and drug naïve patients for each doublet sequence of microstate. Positive value indicates higher occurrence ratio in patients than controls. No significance was demonstrated using double-ended t-tests. (B) T values for each triplet sequence of microstate. Positive value indicates higher occurrence ratio in patients than controls. Dashed lines indicate significance level (p = .05) of the 2-tailed t-test. * p<.05 using double-ended t-test.

T values of all doublet sequences of microstate between two groups are shown in Fig. 3A. No significance was demonstrated in any doublet sequence pattern. T values of all triplet sequences of microstate between two groups are shown in Fig. 3B. Only in the A→C→B sequence, patients group showed significantly lower value (t = −2.47, p = .019). However, a single significant result in 36 tests was below the number expected by chance.

## Discussion

The present results indicate that patients with PD show alterations in a specific subset of sub-second brain functional states. This is consistent with the results from RSN studies using fMRI showing that specific aspects of brain resting networks are affected in patients with PD [25]–[28]. These physiological results are in agreement with the clinical observation that in patients with PD only specific aspects of cognition are affected [42]–[44].

As far as we know, there was only one previous study on spontaneous EEG microstates in PD [45], and microstate clustering was not used in that study. They reported an overall change of microstate lateralization with a more right-anterior to left-posterior orientation in patients with PD, which is consistent with our result of more percent total time in microstates of class A. On the other hand, they reported shorter overall microstate durations in patients, which was inconsistent with our results. This may be explained by considerable methodological differences: They used an eyes-open condition for the resting EEG, whereas we used an eyes-closed condition. In addition, they excluded microstates with a single GFP-peak in the calculation of the average microstate duration, whereas we included them.

Recent studies using simultaneous EEG and fMRI recording demonstrated that EEG-defined microstates correlate significantly with RSNs assessed by fMRI [18], [33]. One of the EEG microstates (identical with class A in the present study) was reported to be correlated with negative blood-oxygen-level dependence (BOLD) activations primarily in bilateral superior and middle temporal gyri [18], areas that are implicated in phonological processing in RSNs [19]-[20]. Thus, more percent total time in microstates of class A in the present study may suggest an abnormality in the temporal lobe, which would not be specific for PD but which has been reported in previous structural studies [46]–[47] or in functional studies of the auditory system [48]–[49] in patients with PD.

More percent total time in microstates of class A has also been reported in patients with schizophrenia [36], [50]. A previous study hypothesized that anxiety is an integral part of the development of schizophrenia in a significant sub-group of cases [51], and anxiety (although unspecific) is the most common symptom in schizophrenia. One may therefore speculate that there is a common neurophysiological trait or state in these disorders that is reflected by an increase in percent total time in microstates of class A.

In the present study, we also demonstrated fewer occurrences in microstates of class C in patients with PD. In a previous study, class C was a predominant microstate class in healthy adult subjects recorded during eyes closed relaxation [29], associating class C with a relaxed idling state. The increased arousal patients may thus reduce the occurrence of microstates of class C. In another study by Mueller et al (2005), the occurrence of microstates of class C were associated with the break-down of a top-down driven illusionary motion, and their occurrence was reduced during periods when a new percept was constructed [52]. One may therefore speculate that the reduction of the number of microstates of class C relates to a difficulty of the patients to re-consider a previously made cognitive-emotional evaluation of some environmental stimuli.

A recent study demonstrated that one of the EEG microstates (identical with class C in the present study) was correlated with positive BOLD activations in the posterior part of the anterior cingulate cortex as well as bilateral inferior frontal gyri, the right anterior insula and the left claustrum [18]. These areas roughly correspond to RSN ‘6’ in a previous study [20]. This RSN includes the fronto-insular cortex which has been found to be part of the saliency network [22], [53] and plays a critical role in switching between central-executive functions and the default mode [54]. What's interesting is that in patient with generalized anxiety disorder [25] and social anxiety disorder [26], recent pioneering studies demonstrated decreased resting state activity in regions that correspond to this saliency network. Our result also suggests a decreased functional connectivity between the insulae and other brain systems during resting conditions in patient with PD or other anxiety disorders. Resting state connectivity between the anterior insulae and the anterior cingulate system was reported to integrate interoceptive information with emotional salience to form a subjective representation of the body [55], and aberration in this system may lead patients with PD to the catastrophic misinterpretation of bodily sensations even during the inter-attack conditions.

In microstate class D, there was no significant difference between patients with PD and normal controls. In previous studies, patients with schizophrenia typically showed shortening of microstates of class D [35]–[36], [50]. A recent study demonstrated that one of the EEG microstate (identical with class D in the present study) was correlated with negative BOLD signal in right-lateralized dorsal and ventral areas of frontal and parietal cortex [18]. These areas correspond to RSN ‘2’ in the study by [20] and this RSN has been reported to be involved in attention [56]. In patients with schizophrenia, various cognitive dysfunctions including attentional deficits have been reported [57]. On the contrary, in patients with PD, cognitive functions are relatively intact [58], while selective aberrant processing for threatening stimuli has been reported in PD [59]. These preserved cognitive abilities in PD may correlate with the normal properties of microstate class D in the present study.

The present article also examined the microstate syntax in patients with PD. A recent study demonstrated a deviant microstate syntax in patients with schizophrenia [50], which suggests abnormalities in the sequence of information processing. We found no robust deviations of microstate syntax in patients with PD, both in doublet and triplet sequences, except for a decreased sequence of A→C→B that may be related to the low occurrence of class C in PD.

### Limitations

There are some limitations of this study. (1) Small number of subjects included in the analysis. (2) Patients and controls were matched for gender and age, but not for socioeconomic status or parental educational history. (3) For a more comprehensive coverage of the severity of panic disorder, more detailed scales (e.g., Panic Disorder Severity Scale [60]) should have been used for the clinical assessment.

### Conclusions

If we accept the concept that microstates are candidates for the “atoms of thought” [31], the deviant thoughts such as catastrophic thoughts [4], [61]–[62] or their anticipatory anxiety about having additional attacks [63] could be reflected in their deviant microstates profiles. The present study has shown that the occurrence of sub-second, transiently stable brain states in resting-state EEG, differs between patients with PD and controls. In the patients, the probabilities of specific states of the active neural networks are thus different already prior to the percept of a stimulus. The interpretation of a stimulus and eventually the selection of a behavioral response are determined by the interaction of pre-activated networks with the activity elicited by the stimulus. Abnormal internal states may therefore be precursors to the abnormal responses observed in the patients. Further studies including the treatment effect are needed to determine whether these findings result from state dependent brain conditions or from brain vulnerability (state-like phenomenon) to PD.
